# Cationic Amphiphilic Drugs Are Potent Inhibitors of Yeast Sporulation

**DOI:** 10.1371/journal.pone.0042853

**Published:** 2012-08-08

**Authors:** Ulrich Schlecht, Robert P. St. Onge, Thomas Walther, Jean-Marie François, Ronald W. Davis

**Affiliations:** 1 Stanford Genome Technology Center, Stanford University, Palo Alto, California, United States of America; 2 Laboratoire d'Ingénierie des Systèmes Biologiques et Procédés, University of Toulouse, Toulouse, France; Texas A&M University, United States of America

## Abstract

Meiosis is a highly regulated developmental process that occurs in all eukaryotes that engage in sexual reproduction. Previous epidemiological work shows that male and female infertility is rising and environmental factors, including pollutants such as organic solvents, are thought to play a role in this phenomenon. To better understand how organic compounds interfere with meiotic development, the model organism *Saccharomyces cerevisiae* was exposed to 446 bioactive molecules while undergoing meiotic development, and sporulation efficiency was quantified employing two different high-throughput assays. 12 chemicals were identified that strongly inhibited spore formation but did not interfere with vegetative growth. Many of these chemicals are known to bind to monoamine-receptors in higher eukaryotes and are cationic amphiphilic drugs. A detailed analysis of one of these drugs, tripelennamine, revealed that it induces sporulation-specific cytotoxicity and a strong inhibition of meiotic M phase. The drug, however, only mildly interfered with pre-meiotic DNA synthesis and the early meiotic transcriptional program. Chemical-genomic screening identified genes involved in autophagy as hypersensitive to tripelennamine. In addition, we found that growing and sporulating yeast cells heterozygous for the aminophospholipid translocase, *NEO1,* are haploinsufficient in the presence of the drug.

## Introduction

Meiosis is a key developmental process that occurs in all sexually reproducing eukaryotes, including unicellular organisms, such as the budding yeast *Saccharomyces cerevisiae*. It gives rise to genetic diversity through homologous recombination between parental DNA, and it keeps chromosome numbers constant from generation to generation by creating haploid gametes. Various studies have indicated that environmental factors, such as organic solvents, heavy metals, or heat can negatively impact gametogenesis in man [1], [2]. It remains unclear, however, to what extend exposure to organic compounds (*e.g.* drugs) can lead to infertility, and which specific stages of meiotic development are compromised. Such studies are difficult to conduct in humans due to ethical issues and therefore the development of experimental systems using model organisms would be beneficial.

Meiosis and sporulation in yeast and spermatogenesis in higher eukaryotes are analogous developmental pathways. Characteristic landmark events including pre-meiotic DNA synthesis, recombination, and chromosome segregation during the first and second meiotic divisions (MI and MII) are controlled in a highly similar fashion and rely on conserved genes, many of which display transcriptional up-regulation during these processes [3], [4], [5]. These developmental stages are followed by morphogenetic differentiation events, which give rise to the formation of functional haploid gametes (commonly referred to as spores in budding yeast).

Numerous studies have demonstrated that meiotic development in yeast is coordinated at several levels including signal transduction [6], transcriptional regulation [7], [8], meiosis-specific splicing [9], [10], mRNA turnover [11], post-translational modification [12] and degradation [13] of regulatory proteins. Two nucleus-associated structures, the synaptonemal complex and the spindle-pole bodies, play important roles in coordinating proper reciprocal exchange between the homologous chromosomes during MI and packaging of meiotic products into mature gametes (reviewed in [14]).

In addition, sporulation in yeast is also regulated on a metabolic level. In budding yeast meiotic development is induced when vegetative cells are transferred to a nitrogen-free medium containing acetate as the sole carbon source (reviewed in [15]). Sporulating yeast cells undergo strong physiological changes, including a decrease in RNA and protein content, an accumulation of the storage carbohydrates [16] and spore wall components [17], and a large increase in oxygen consumption. Because of the absence of external nitrogen sources, 60–70% of the pre-existing vegetative protein is degraded to generate a supply of amino acids essential for the synthesis of new sporulation-specific proteins [18].

Despite the aforementioned wealth of data available for regulatory mechanisms governing yeast meiosis and sporulation, currently only little is known about small molecules that have the potential to interfere with these processes. Early studies demonstrated that nitrogen-containing compounds, such as amino acids and ammonium ions prevent yeast cells from sporulating [19]. Other work described the effects of chemicals that induce aneuploidy in yeast undergoing meiosis [20]. Anti-neoplastic drugs, such as adriamycin, mitomycin C, and bleomycin were shown to disrupt the second meiotic division leading to the generation of diploid spores [20]. These drugs, however, are not only effective during sporulation, but also abolish vegetative growth.

In this study we aimed to identify chemicals that inhibit meiotic development in yeast but do not interfere with vegetative growth. We profiled a library of 446 drugs from the NIH clinical collection with two sporulation assays, and generated sensitivity profiles of growing and sporulating cells for each of these chemicals. This approach identified 12 potent, sporulation-specific inhibitors, the majority of which are cationic amphiphilic drugs. We have studied the effects of one of these drugs, tripelennamine, on various meiotic landmarks and identified genes related to autophagy as hypersensitive to the drug using chemical genomic profiling.

## Results

### Two Assays for the Identification of Chemical Inhibitors of Sporulation

**Figure 1:**
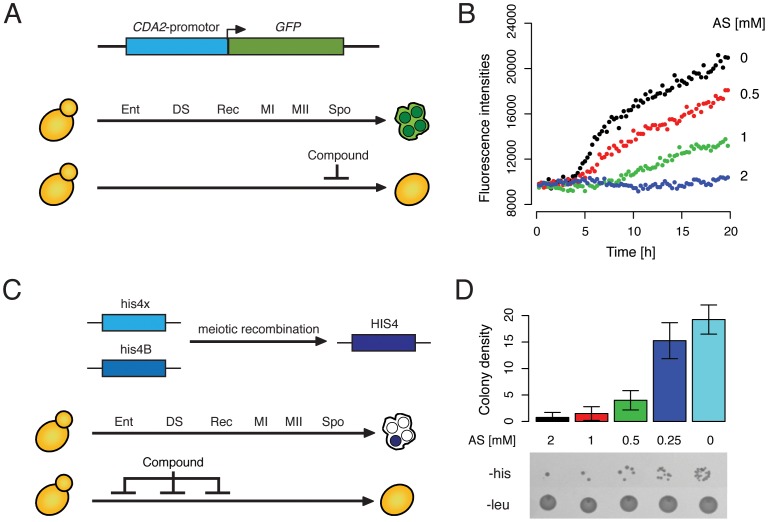
Two screening assays for sporulation efficiency. (A) Schematic of the fluorescence-based assay used to identify sporulation inhibitors. Meiotic landmark events indicated as Ent (entry), DS (pre-meiotic DNA replication), Rec (recombination), MI and MII (first and second meiotic division), and Spo (spore formation). The normal final product is depicted as an ascus with four spores (green). Compounds that inhibit sporulation or that are cytotoxic in sporulating yeast cells will suppress the expression of the sporulation-specific gene *CDA2* and therefore also *CDA2* promotor-driven *GFP* expression. (B) Real-time measurement of fluorescence intensities in cells harboring the *GFP*-reporter, which were sporulated in the presence of varying concentrations of ammonium sulfate (AS) as indicated. Fluorescence of the sporulation culture was measured every 15 min over the course of 20 hours (see Methods). (C) Schematic of the post-recombination growth assay. The two defective alleles (*his4x* and *his4B*) can give rise to a functional *HIS4* allele upon meiotic recombination. The histidine-auxotrophic diploid mother cells can therefore produce histidine-prototrophic spores (indicated in blue). If this event is suppressed by a compound, either because it directly inhibits meiotic recombination or because it is cytotoxic to sporulating yeast cells, no histidine-prototrophs are formed. (D) Proof-of-concept of the *his4x*/*his4B* assay. Cells were sporulated for 5 hours in the presence of varying concentrations of ammonium sulfate (AS) as indicated. Aliquots of cells were then transferred to agar plates that lack histidine (-his) or leucine (-leu) and incubated for two days at 30°C, prior to measuring colony density (see Methods) of each spot on the -his agar. Agar plates lacking leucine served as a loading control. The barplot in the upper panel shows mean and standard deviation data from 4 independent experiments.

To monitor sporulation efficiency, a fluorescence-based microtiter plate assay was developed. The transcription of *CDA2*, a sporulation-specific chitin deacetylase involved in the biosynthesis of the spore wall component chitosan [21], was used as a read-out in this assay. Previous meiotic expression profiling analyses showed that mRNA levels of *CDA2* are not detectable in vegetative cells but strongly increase in the middle period of sporulation, with peak expression during spore wall formation (corresponding to ∼6 to 10 hours of sporulation in SK1, the strain background used in this study) [7], [22]. To measure the transcriptional activity of the *CDA2* locus in hundreds of different chemical treatment conditions we constructed a plasmid that encodes *eGFP* (enhanced green fluorescence protein) under the control of the *CDA2*-promotor (Figure 1A). We transformed SK1 with this plasmid and monitored *GFP* expression in real-time using a Tecan Safire, a fully modular monochromator-based detection system. Steadily increasing fluorescence signals were detected starting at 5 hours after transfer into sporulation media (Figure 1B). To test the sensitivity of this detection system we added varying concentrations of ammonium sulfate, which is known to inhibit entry into meiosis in budding yeast [19] by suppressing the expression of *IME1* [23]. As expected, expression of *GFP* was suppressed by ammonium sulfate in a concentration-dependent manner (Figure 1B). When present at 2 mM in the sporulation media, ammonium sulfate completely repressed *GFP* expression. Lower concentrations (1 mM and 0.5 mM) allowed a fraction of the cells to undergo spore formation. Decreasing fluorescence intensities were indicative of decreasing sporulation efficiency as determined by microscopy (0%, 13%, 56%, and 98% spores were observed after 24 hours at 2 mM, 1 mM, 0.5 mM and 0 mM ammonium sulfate, respectively). These results indicated that our assay can identify chemical compounds that inhibited sporulation via their effect on *CDA2* expression (Figure 1A).

Previous analyses of meiotic mutants in yeast have shown that cells can omit certain stages of meiotic development and still produce mature meiotic products (reviewed in [14]). For example *spo11*Δ mutants, that are unable to perform meiotic recombination, are still capable of producing mature asci. Thus, chemical compounds that for example inhibit *Spo11* would not be identified with the fluorescence-based assay described above. To overcome this limitation a second screening approach was employed. This approach is based on a hetero-allelic reporter system that has been used by others to measure meiotic reciprocal recombination [24], crossover and non-crossover recombination [25], and recombination frequencies [7]. A strain harboring the *his4* mutant alleles (*his4x* and *his4B*) is unable to grow in the absence of histidine. Upon meiotic recombination between the two alleles, one of the four meiotic products will receive a functional *HIS4* allele, generating a histidine-prototrophic cell that is capable of growing in the absence of histidine. This event is facilitated by the presence of two recombination hot-spots located within the *HIS4* open-reading frame [26]. The production of histidine-prototrophs can be monitored by transferring aliquots of sporulating cells to media lacking histidine. Compounds that inhibit entry into meiosis, pre-meiotic DNA replication or recombination will suppress recombination between the *his4* alleles and will therefore suppress the generation of such prototrophs (Figure 1C). To validate this reporter assay, a proof-of-concept experiment was performed in which different concentrations of ammonium sulfate were added to his4x/his4B harboring cells upon induction of meiosis (Figure 1D). After 5 hours of sporulation, where most cells have undergone pre-meiotic DNA-synthesis and meiotic recombination but have not undergone the commitment and can therefore return to growth [27], aliquots of the cultures were plated onto agar plates lacking histidine. As expected, the quantity of histidine-prototrophic cells increased with decreasing concentrations of ammonium sulfate in the media (Figure 1D). Results from this assay correlated with those from the fluorescence-based assay: 2, 1, and 0.5 mM of ammonium sulfate suppressed colony formation (Figure 1B); lower concentrations of ammonium sulfate did not interfere with meiotic recombination and hence colony growth (Figure 1D). Note, that in addition to compounds that specifically inhibit meiotic recombination and/or spore formation, the two screening assays described here will also identify compounds that are cytotoxic in cells undergoing these processes. Taken together, these are complementary approaches to screen for sporulation-inhibiting compounds or compounds that are cytotoxic in sporulating yeast cells.

### Identification of Sporulation-specific Inhibitors

The US National Institutes of Health Clinical Collection (NCC) was used as a source of chemical compounds. This library comprises 446 compounds used in human clinical trials. We first decided to identify compounds that negatively affect vegetative growth of yeast. To this end we determined growth rates of a wild-type (BY4741, [28]) and a mutant strain that lacks 9 of the major drug-efflux pumps (AD1-9, [29]) in the presence of each compound from the NCC. For each chemical, a sensitivity score was calculated (see Methods) based on the change in growth rate in response to chemical treatment compared to “no drug” controls. The growth rates of BY4741 and AD1-9 in the presence of all compounds tested are depicted in Figure S1. As expected, growth of the drug-efflux pump deficient strain was more often and more strongly inhibited than that of the wild-type strain. Altogether, 231 compounds inhibited growth of BY4741 and/or AD1-9.

**Figure 2:**
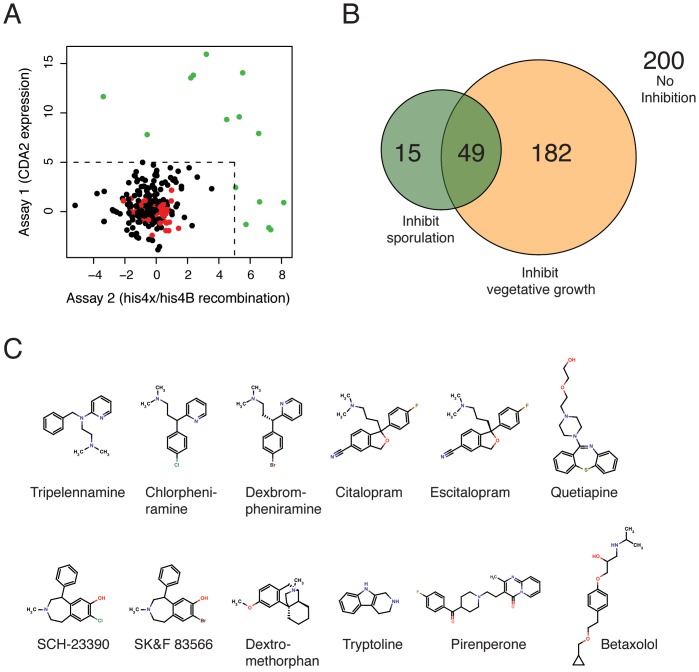
Small molecule inhibitors of yeast sporulation. (A) A scatterplot of sensitivity scores (calculated as described in the Methods) representing the impact of compounds from the NIH clinical collection in the sporulation screening assays. Compounds that inhibit growth or that interfered with the fluorescence-based assay because of auto-fluorescence were not included in this representation. Compounds with a score of greater than 5 are shown in green. DMSO controls are shown in red. (B) A Venn diagram representing the overlap of inhibitors in the growth assay and the two sporulation assays (colored in yellow and green, respectively). 200 compounds had no effect in any of the three assays, 231 inhibited vegetative growth (of BY4741 and/or AD1-9 strains), and 64 inhibited sporulation. The overlap of between growth and sporulation inhibitors was 49. (C) Chemical structures of 12 sporulation-specific inhibitors that were confirmed to inhibit sporulation by microscopy analysis.

To identify meiosis-specific inhibitors, all drugs in the NCC were subsequently interrogated with the two sporulation assays. For these experiments we used the efficiently-sporulating SK1 strain background, which was not deficient in any of the drug-efflux pumps. Similar to the growth data, we calculated sensitivity scores for every compound. This score indicates how strongly a compound inhibited sporulation in the assay compared to the “no drug” controls. To minimize false positives and prioritize secondary experiments, a highly stringent cutoff was applied (see Methods). A scatterplot depicts the sensitivity scores determined this way (Figure 2A) and a Venn diagram summarizes the overlap of compounds identified in the three different screening approaches (Figure 2B). In summary, 200 drugs had no effect in any of the assays; 231 inhibited growth and 64 inhibited sporulation. 49 drugs inhibited vegetative growth and sporulation. All sensitivity scores are listed in Table S1.

15 compounds were found to specifically inhibit spore formation (Figure 2B and Table 2). We re-tested these chemicals in sporulating cultures and determined the percentage of spores after 48 hours. Note that these experiments were performed in small liquid cultures (300 µl), which are sub-optimally aerated, and thus only intermediate sporulation efficiencies were observed (*e.g.* 65% in the “no drug” control). Nevertheless, 10 of the 15 compounds completely abolished spore formation and 2 chemicals (Tryptoline and Pirenperone) had moderate inhibitory effects. The remaining 3 compounds were indistinguishable from the control and were not further analyzed. Taken together, the confirmation rate was 80% (12 out of 15 compounds initially identified in our screen). Five of the twelve compounds (Betaxolol, SCH-23390, SKF 83566, Tryptoline, Pirenperone) were identified in the sporulation assay only. Another set of 5 drugs (Chlorpheniramine, Citalopram, Dexbrompheniramine, Escitalopram, Tripelennamine) was identified in the recombination assay only. The remaining 2 compounds (Dextromethorphan and Quetiapine) were identified in both assays (Table 2).

We noticed a similarity in molecular structures among the inhibitory compounds: all contained a hydrophobic ring system and a basic nitrogen-containing group (Figure 2C), which are the attributes of a class of compounds called cationic amphiphilic drugs. The primary targets of these drugs in man are monoamine receptors, and many are widely used anti-depressants and anti-psychotics (*e.g.* Citalopram, Quetiapine, Tripelennamine). These drugs are not only known to interact with their protein targets, but also with phospholipid membranes. Yeast lack proteins with sequence similarity to monoamine receptors, therefore, these drugs likely repress sporulation by inhibiting alternative proteins or cellular components (*e.g.* phospholipid membranes). We chose tripelennamine (TA) as a representative for this class of compounds, and studied its effect on yeast sporulation in more detail.

### Tripelennamine Reduces Viability of Sporulating Yeast and Inhibits Meiotic M-phase

**Figure 3:**
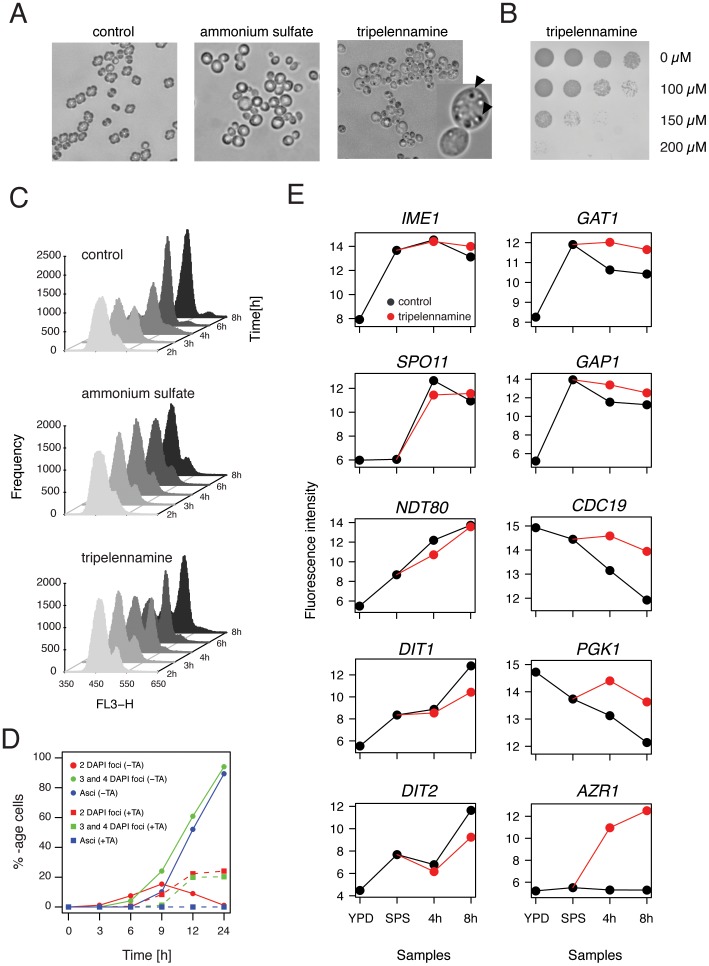
Tripelennamine strongly inhibits viability and meiotic M-phase in sporulating yeast. (A) Images of Nomarski microscopy of cells sporulated in the absence of drug (control), in the presence of 2 mM ammonium sulfate (AS), or 100 µM tripelennamine (TA) for 24 hours. Part of the TA image was magnified; arrows indicate granular bodies of unknown origin. (B) 5-fold serial dilution of yeast sporulated for 24 hours in the presence of three different concentrations of TA (indicated on the right) and then transferred to rich media in order to determine the rate of cell survival. Pictures were taken after incubating the rich media agar plates for 2 days at 30°C. (C) FACS analysis of pre-meiotic DNA synthesis of a “no drug” control, and in cells treated with ammonium sulfate (2 mM), or tripelennamine (100 µM). Samples were taken at 2, 3, 4, 6, and 8 hours after induction of sporulation. Staggered histograms show the frequencies (plotted on the y-axis) of relative DNA content, measured as propidium iodide intensity (plotted on the x-axis). (D) Percentage of cells that have completed meiotic M-phase (cells with 2 DAPI foci and cells with 3 or 4 DAPI foci) and those that have formed mature asci over time in sporulation medium (given in hours, x-axis) in the absence (control, circles and solid lines), or the presence of 100 µM tripelennamine (treatment, squares and dashed lines). (E) Expression patterns of representative genes involved in meiotic development (left column), nitrogen catabolite repression (*GAT1* and *GAP1*), glycolysis/gluconeogenesis (*CDC19* and *PGK1*), and stress response (*AZR1*). Log2-transformed fluorescence signals of RMA-normalized microarray data (see Methods) are plotted on the y-axis and are graphed versus samples taken in rich media and pre-sporulation media (in the absence of tripelennamine), or total time (4 and 8 hours) the cultures spent in sporulation media in the absence (black curve) or presence (red curve) of TA.

We first determined sporulation efficiency in the presence of TA in large (100 ml) liquid cultures. As expected the “no drug” control yielded ∼95% mature spores within the first 24 hours, and ammonium sulfate strongly reduced spore formation to only about 10% after 3 days. In contrast, TA completely abolished sporulation at a concentration of 100 µM. Even after 72 hours no spores were detected in the TA-treated culture. Upon visual inspection of sporulating cultures by microscopy, marked morphological differences were observed in these three conditions (Figure 3A). In the “no drug” control, most cells had formed an ascus with spores, whereas the ammonium sulfate-treated cells had round and inflated shapes. In contrast, cells that were sporulated in the presence of TA had accumulated small granular bodies of unknown nature, but were devoid of spores. When aliquots of this culture were transferred onto YPD plates, we discovered that chemical treatment during sporulation had a cytotoxic effect (Figure 3B). This effect was concentration-dependent: while treatment with 100 µM of TA was only mildly toxic to a sporulating culture, at 200 µM almost all sporulating cells were inviable. In contrast, TA had no effect on vegetative cultures grown in rich and minimal media at these concentrations (Figure S2).

To better determine the timing of TA-inhibition during sporulation, we performed two types of time-course experiments. In the first we added TA to the sporulation media at various time-points after induction of meiosis and determined the percentage of sporulated cells after 24 hours. Addition at 3 or 6 hours strongly inhibited spore formation: 4% and 28% spores, respectively (Figure S3). In contrast when added after 9 or 12 hours, sporulation efficiency was comparable to that of an untreated culture: 84% and 92%, respectively. To complement these data we also performed a second time-course experiment, where TA was added to the sporulation culture at the onset of meiosis, and was then washed out of the media at various time-points after induction of meiosis. When the drug was removed from the sporulation media at 0 or 3 hours, a high spore count was measured: 90% and 83%, respectively. Removing the drug after 6 hours resulted in an intermediate efficiency of 48%. Cells in which the drug was removed at the later time-points (9 and 12 hours) underwent sporulation with very low frequencies. From this we conclude that the window of inhibitory activity of TA is between 3 and 9 hours, which corresponds to the timing of meiotic recombination and chromosome segregation in SK1.

We next asked whether TA prevents cells from undergoing meiotic S-phase. To this end, pre-meiotic DNA synthesis was monitored by flow cytometry at 2, 3, 4, 6, and 8 hours after transfer into sporulation media (SPII) (Figure 3C). As expected, in the absence of drug-treatment the process was completed within the first 6 hours and ammonium sulfate-treated cells did not initiate pre-meiotic DNA synthesis within the time-window monitored here. In contrast, TA only mildly interfered with pre-meiotic DNA synthesis. In the presence of the drug, the majority of the cells had completed the process after 8 hours (Figure 3C). We next tested the impact of TA on meiotic M phase. DAPI staining of nuclei revealed that the appearance of bi- and tetranucleate cells (between 6 and 12 hours) was strongly reduced in TA (Figure 3D). Only about 40% of drug-treated cells had proceeded through at least one of the two divisions within 24 hours. In comparison, in the “no drug” control, almost all cells had undergone both meiotic divisions within 12 hours of sporulation. Taken together these data suggest that TA does not interfere with pre-meiotic DNA synthesis but strongly inhibits meiotic M phase.

Next we examined whether TA interferes with transcription of meiotic genes. In yeast, a highly coordinated transcriptional program is initiated upon entry into sporulation (reviewed in [15], [30]) that involves the transient up-regulation of approximately 900 genes [7]. About ∼20% of these transcripts are essential for the process [31], [32], [33], [34]. This includes genes involved in pre-meiotic DNA synthesis, recombination of homologous chromosomes, the establishment of the synaptonemal complex, the completion of M phase, and spore morphogenesis. To determine whether TA changes the meiotic transcriptional program, a global gene expression profiling experiment was performed. For this experiment we collected samples of SK1 growing in rich media (YPD) and pre-sporulation media (SPS) in the absence of TA. We then transferred the culture to sporulation media, split it into a “no drug” and a TA-treatment culture, and harvested samples at 4 and 8 hours after induction of meiosis. RNA was extracted from these samples and analyzed using Affymetrix Yeast 2.0 Genechips (Figure S4).

54 genes were up-regulated (2-fold or higher) in TA samples at both 4 and 8 hours when compared to control samples (Figure S4). Surprisingly, none of these loci were directly involved in meiotic development or sporulation. Examples of transcript levels of meiosis-specific genes are depicted in Figure 3E. Notably, no significant difference was observed in the height of expression of *IME1* (initiation of meiosis), *SPO11* (meiotic recombination), *SPO13* (sister chromatid cohesion), and *NDT80* (transcriptional induction of middle meiotic genes) between TA-treated and “no drug” control samples. We noted, however, a reduced expression of *DIT1* and *DIT2*, two sporulation-specific enzymes involved in spore wall maturation (the last step during sporulation) in the presence of TA at the 8 hour time-point. Taken together, this suggested that TA did not inhibit meiotic development by suppressing the expression of meiosis-specific genes that control early and middle meiotic events such as pre-meiotic DNA synthesis and meiotic recombination.

Among the 54 loci that showed higher expression in TA, we found an enrichment of genes involved in glycolysis (*p*-value: 2.91 * 10^−10^) (*PGI1, TDH1, PGK1, GPM1 ENO1, ENO2, CDC19, TYE7*). In addition, numerous genes involved in amino acid metabolism (*ARG1, ADE15, ADE17, HIS4, ILV3, TKL1*), amino acid transport (*AGP1, DIP5, GAP1, ODC2, VBA1*), allantoin metabolism (*DAL5, DAL7, DUR1,2*), and nitrogen catabolite repression (*DCG1, GAT1, GAP1*), also showed higher expression in TA (Figure S4). During sporulation yeast cells shift their metabolism towards respiration, using acetate as a non-fermentable carbon source [35]. Nitrogen is produced through internal degradation of pre-existing proteins [18]. The finding that TA induces genes involved in glucose and nitrogen catabolism suggested that TA changed the metabolic state of the cell.

The strongest induction (>60-fold in TA) was observed for *AZR1*, a gene that encodes a plasma membrane transporter involved in azole drug resistance (Figure 3E) [36]. Although TA is not an azole, it appears to activate multidrug resistance response in yeast. This notion is corroborated by the fact that transcriptional activity of two additional ABC multidrug resistance transporters, PDR5 and SNQ2, are also more abundant in TA (Figure S4). We also found an induction of genes involved in response to stress (*GRE2, YJL144W*) and cell wall damage (*YLR194C, YLR414C*). Only a single gene, *RCK1* (a protein kinase involved in the response to oxidative stress), was found to be down-regulated in the presence of TA.

To address the question how TA-treatment affects levels of metabolites in sporulating cells, a detailed metabolome analysis was carried out comparing TA-treated versus “no drug” control cells. Metabolites were extracted from the cells at 5, 9 and 24 hours after transfer to sporulation medium, and a total of 37 metabolites occurring in glycolysis, TCA and glyoxylate cycle, nucleotide metabolism, and reserve carbohydrate metabolism were quantified by IC-MS analyses (Table S2). After 24 hours exposure to TA, cells were completely devoid of triphosphate nucleotides (ATP, GTP, CTP, UTP) indicating energetic depletion and cell death. However, during an exposure to TA of up to 9 hours, which corresponds to the time window where TA affects sporulation (Figure S3), only mild changes in the observed metabolite pools were detected. Cellular energy levels, *i.e.* ATP concentrations, were indistinguishable between TA-treated and “no drug” control cells ruling out the possibility that TA interferes with respiration and energy supply directly. Furthermore, the concentration of the second messenger cAMP was the same in TA treated and control cells, indicating that the apparent up-regulation of glycolytic genes (Figure S4) was not induced by cAMP-dependent signaling. The absence of significant differences in all measured glycolytic metabolite concentrations argued for non-compromised glycolytic function. The only metabolites that exhibited a more than two fold difference between TA-treated and control cells were citrate, isocitrate, and α-ketoglutarate, as well as glycerol-3-phosphate. Glycerol-3-phosphate is at the entry point into lipid and phospholipid metabolism, α-ketoglutarate is closely related to glutamate and thus amino acid metabolism. Differences in the accumulation of these metabolites might be indicative for perturbations in these metabolic pathways. However, this notion remains highly speculative. In conclusion, the metabolome analysis confirmed cell death after 24 hours of exposure to TA, and largely ruled out direct interference of glycolysis, nucleotide metabolism, and respiratory metabolism as the mechanism of TA’s inhibitory effect.

### Autophagy-deficient Mutants are Tripelennamine-sensitive

**Figure 4:**
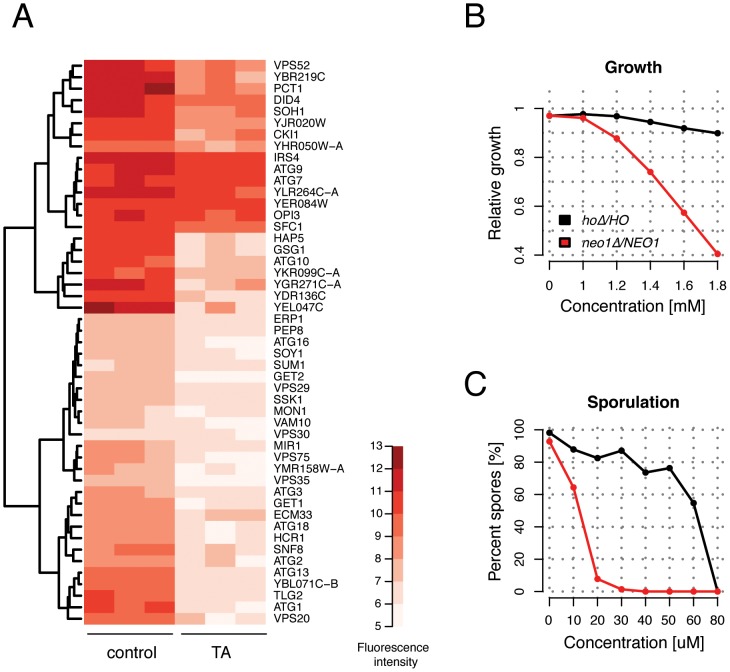
Tripelennamine sensitizes autophagy-deficient yeast mutants. (A) Heatmap and dendrogram depicting hierarchical clustering of homozygous deletion pool data from three independent experiments. Quantile normalized and log2-transformed microarray fluorescence signals were analyzed using the Significance Analysis of Microarrays (SAM) software (see Methods) and identified 49 genes (indicated on the right-hand side) that were significantly depleted in the presence of tripelennamine (TA) compared to a “no drug” control. Darker shades of red indicate higher fluorescence signals and therefore a higher abundance of that strain in the pool (see legend). (B) Growth of the *ho*Δ*/HO* (control) and *neo1*Δ*/NEO1* heterozygous deletion strains (see legend) were determined in the presence of various concentrations of (TA) (indicated on the x-axis). Growth rates relative to the “no drug” control (calculated as a ratio of AvgG, see Methods) were determined for each concentration and are plotted on the y-axis. (C) Sporulation efficiency of the *ho*Δ*/HO* control strain (black curve) and the *neo1*Δ*/NEO1* heterozygous deletion strain (red curve) sporulated in the presence or absence of TA is indicated as percent spores on the y-axis. Both strains were incubated in sporulation media for 48 hours at various concentrations of TA (indicated on the x-axis) and percentage of spores was determined by microscopy. A total of 100 cells were counted for every condition.

To better understand the mode of action of TA, we performed a chemical-genomic screen to identify genes required for resistance to TA [37], [38], [39]. This approach, termed homozygous profiling, is based on the idea that gene deletions that render cells hypersensitive to a specific compound identify pathways that buffer against the toxic effects of that compound and therefore provides clues about the molecule’s activity. This type of pathway-analysis can be performed on a genome-wide scale using the yeast homozygous gene deletion collection, in which both copies of every non-essential gene is deleted in a diploid strain to produce a complete loss-of-function allele. A pool of approximately ∼4600 diploid homozygous deletion strains was incubated in sporulation media in the presence and absence of TA for 3 days (see Methods). This approach will presumably identify genes important for resistance to TA under starvation conditions. A list of 49 strains that were significantly sensitized in TA compared to the “no drug” control was identified (Figure 4A). No deletion strain was found to be more abundant in, or in other words resistant to, TA. To search for enriched functional categories among the group of 49 genes the Gene Ontology Term Finder tool in the Saccharomyces Genome Database was used. A strong over-representation of genes involved in vacuolar transport (*p*-value: 2.96 * 10^−16^) and more specifically autophagy (p-value: 1.19 * 10^−11^) (*ATG1*, *ATG2*, *ATG3*, *ATG7, ATG9*, *ATG10*, *ATG13*, *ATG16*, *ATG18*, *IRS4, MON1, TRS85, VPS30*) was found. These data suggested that a dysfunctional autophagy pathway contributes to TA toxicity. This observation is in line with a recent study demonstrating that yeast cells treated with sertraline, which like TA is a cationic amphiphilic drug, accumulates incompletely digested autophagosomal intermediates [40]. The same study also found that a diploid strain heterozygous for *NEO1* is sensitive to sertraline. *NEO1* is an essential gene that encodes a putative aminophospholipid translocase (or flippase), involved in moving phospholipids from one side of the membrane bilayer to the other [41]. Neo1 is also required for controlling vacuolar pH [42]. To test whether TA induces haploinsufficiency of *NEO1* in growing and/or sporulating cultures, we constructed a *neo1Δ/NEO1* heterozygous mutant in the SK1 background. The strain exhibited an increased sensitivity to tripelennamine compared to the wild-type control strain during vegetative growth (Figure 4B and S5). Similarly, the *neo1Δ/NEO1* heterozygous strain was significantly more sensitive to TA exposure during sporulation than the control strain, exhibiting severely reduced sporulation efficiency at TA concentrations as low as 10–20 µM (Figure 4C). These data indicate that TA-dependent inhibition of sporulation is enhanced by reduced Neo1 activity.

## Discussion

In the present study two assays were developed that quantify sporulation efficiency, and thus enabled us to identify small molecule inhibitors of spore formation in budding yeast. We applied these assays to measure sporulation efficiency in response to treatment with 446 drugs that have been tested in human clinical trials for a wide variety of therapeutic indications. Out of these, 12 were identified that inhibited meiotic development, but not vegetative growth. Strikingly, these sporulation-specific inhibitors were structurally related to a class of compounds called cationic amphiphilic drugs, or CADs. Members of this class are weak bases with lipophilic properties, and tend to accumulate in acidic intracellular compartments such as lysosomes. Once inside the acidic milieu of the lysosome, the molecules become protonated, can no longer permeate the membrane and get trapped inside the organelle, a phenomenon referred to as lysosomotropism. Ultimately, the excess accumulation of CADs can give rise to a lysosomal storage disorder, called phospholipidosis [43]. Hallmarks of phospholipidosis are the formation of multilamellar vesicles that can lead to the disruption of organelle integrity and an alteration of phospholipid metabolism. Recent work demonstrated that the anti-depressant CAD sertraline evokes phenotypes in yeast that resemble those of phospholipidosis [40]. Cationic amphiphiles have also been shown to interfere with the process of autophagy. During autophagy, cytoplasmic cargo is captured into autophagosomes, a double membraned vesicle, followed by fusion of the autophagosome with the lysosome/vacuole to form an autolysosome where the captured material is degraded (reviewed in [44], [45]). The anti-malarial drug chloroquine, a CAD, has been shown to accumulate inside autophagic vacuoles and to increase the intralysosomal pH [46]. This inhibits the acid-dependent degradation of autophagosome content and results in the accumulation of autophagic vesicles that cannot be cleared from the cytoplasm [47]. Similarly, yeast cells treated with sertraline, appeared to contain large inclusions of incompletely digested autophagosomes and vacuoles exhibiting increased electron-transparency, suggesting a loss of vacuole acidity and/or impaired delivery of vacuolar hydrolases [40]. In yeast the limitation for any of the essential nutrients can trigger autophagy, with nitrogen limitation displaying the strongest stimulus [48]. In the absence of external nitrogen sources, yeast defective in autophagy experience a strong depletion of internal amino acids, which precludes the synthesis of proteins important for surviving nitrogen starvation and can result in accelerated cell death [49]. Autophagy therefore provides the primary source of nitrogen under starvation condition. This is presumably also the case during sporulation, a process that is induced in yeast when external nutrients are lacking. Indeed, several studies have demonstrated that autophagy is essential to sporulating cells [31], [32], [33].

Several observations made in the present study support a model in which TA could inhibit sporulation by interfering with autophagy. First, the chemical-genomic screen with the homozygous deletion collection identified autophagy-related mutants as hypersensitive to TA. Interestingly, some of the genes that were identified in this screen are involved in autophagosome formation, such as *ATG2*, *ATG9*, or *ATG18* [50], [51], [52]. A possible interpretation of our screening data is therefore, that a non-essential pathway that functions in parallel to autophagosome formation, for example its fusion with the lysosome, is affected by TA. Second, transcription of genes involved in amino acid metabolism and transport were found to be up-regulated in the presence of the drug, suggesting that the cells are experiencing a lack of internal amino acids. Third, it is tempting to speculate that the granular structures that were observed in TA-treated sporulating yeast (Figure 3A) are undigested autophagosomes. Fourth, a recent study showed that the deletion of *CCZ1*, a gene important for both sporulation and autophagy, exhibits phenotypes similar to those observed in TA-treated yeast undergoing sporulation: while the meiotic transcriptional program and pre-meiotic DNA synthesis were largely unchanged, the meiotic divisions were strongly inhibited [53]. Finally, we found that the *neo1*Δ*/NEO1* heterozygous strain is highly sensitized to TA in sporulating cultures. Neo1 is involved in intracellular membrane-trafficking, protein sorting and vacuole biogenesis. Temperature-sensitive mutants of neo1 have been shown to exhibit fragmented and hyper-acidic vacuoles [42], [54]. Thus, the observed sensitivity of the *neo1*Δ*/NEO1* strain to TA may be due to decreased vacuolar pH of the strain, resulting in elevated trapping of TA in the vacuole and increased obstruction of autophagy. Additional work is needed to elucidate the precise mechanism by which TA suppresses spore formation and to determine to what extent autophagic processes are involved. Since all 12 drugs identified here are positively charged amphiphiles it is tempting to speculate that they have a common mechanism of action. Further experimentation will however be necessary to test this hypothesis. In summary, we have found that cationic amphiphilic drugs are potent inhibitors of yeast sporulation. The data presented here open up an important avenue to study metabolic and membrane processes required for sporulation in yeast.

## Materials and Methods

### Yeast Strains and Genetic Manipulations

Table 1 lists yeast strains used in this study. Yeast media were prepared and genetic methods were carried out as described by [55]. Yeast strains were transformed using the lithium acetate procedure described in [56]. The following media were used for growth and sporulation of *Saccharomyces cerevisiae*: rich media YPD (1% yeast extract, 2% bactopeptone, 2% glucose), pre-sporulation media SPS (0.5% ammonium sulfate, 0.17% yeast nitrogen base, 1% bactopeptone, 1% potassium acetate, 0.5% yeast extract, 1.02% potassium hydrogen phthalate, set pH to 5.5 with 10 N potassium hydroxide solution), sporulation media SPII (2% potassium acetate). Complete synthetic media lacking histidine (-his) or leucine (-leu) contained 0.17% yeast nitrogen base, 2% glucose, 0.5% ammonium sulfate, and appropriate amino acid drop-out mixtures.

**Table 1:** Strains used in this study.

Strains	Genotype	Source
USY61	MATa/MATalpha ura3Δ0/ura3Δ0 his3Δ1/his3Δ1 CAN1/can1::Ste2::spHis5 flo8Δ0/flo8Δ0	gift from Adam Deutschbauer
USY613	USY61+ pCDA2-eGFP::HygB	This study
USY1634	USY61+ neo1::kanMX/NEO1	This study
NKY1551	MATa/MATalpha ho::LYS2/ho::LYS2 ura3/ura3 lys2/lys2 leu2::hisG/leu2::hisG arg4-Nsp/arg4-Bgl his4x::LEU2-URA3/his4B::LEU2	[7]
BY4741	MATa his3Δ0 leu2Δ0 met15Δ0 ura3Δ0	[28]
AD1–9	MATalpha, pdr1–3, ura3, his1, Δyor1::hisG, Δsnq2::hisG, pdr5-Δ2::hisG, Δpdr10::hisG, Δpdr11::hisG, Δycf1::hisG, pdr3-Δ2::hisG, Δpdr15::hisG, pdr1-Δ3::hisG	[29]

**Table 2:** Verification of sporulation-specific inhibitors identified in this study.

Name	Pubchem	Percent spores	Inhibition
	identifier	after 48 h	class
Betaxolol	CPD000058420	0	CDA2
Chlorpheniramine	CPD000466271	0	HIS4
Citalopram	CPD000326936	0	HIS4
Dexbrompheniramine	CPD000471616	0	HIS4
Dextromethorphan	CPD000326694	0	CDA2 and HIS4
Escitalopram	CPD000469191	0	HIS4
Quetiapine	CPD000471623	0	CDA2 and HIS4
SCH-23390	CPD000326935	0	CDA2
SKF 83566	CPD000449276	0	CDA2
Tripelennamine	CPD000058623	0	HIS4
Tryptoline	CPD000059115	25	CDA2
Pirenperone	CPD000058507	34	CDA2
Tiagabine	CPD000469176	56	HIS4
SR 57227A	CPD000449299	61	CDA2
Nitazoxanide	CPD000466367	64	CDA2 and HIS4
“no drug” control	NA	65	NA

### Real-time Measurement of GFP Signals in Sporulating Yeast Cells

A fresh yeast colony was inoculated into 5 ml YPD (supplemented with Hygromycin B to maintain the pCDA2-eGFP harboring plasmid) and grown over night to saturation. 200 µl of this cell culture were inoculated into 30 ml of SPS (supplemented with Hygromycin B) and grown until an optical density at 600 nm between 1.2 and 1.6 was reached. The cell suspension was centrifuged for 3 min at 3000 rpm and washed twice with 50 ml of pre-warmed water. Finally, the cell pellet was resuspended in 50 ml SPII. 100 µl of this sporulation culture was pipetted into each of the wells of a 96 Well Assay Plate (Cat. No. 3603 from Corning Inc). Chemical compound or DMSO (“no drug” control) was then added to the wells at a 1∶100 dilution; the stock concentration of each compound tested is listed in Table S1. The Assay Plate was covered with a gas permeable sealing membrane (Cat. No. 9123-6100 from Diversified Biotech). A Safire2 microplate reader (Tecan Systems Inc. San Jose, USA) was used to measure sporulation-specific expression of GFP using the following settings: Excitation Wavelength: 488 nm (bandwidth 100); Emission Wavelength: 520 nm (bandwidth 100); the number of multilabel kinetic cycles and intervals were 95 and 900 sec, respectively.

### Yeast Post-germination Growth Assay

A fresh colony of a diploid yeast culture harboring the heteroallelic reporter system (his4x/his4B) was inoculated into 5 ml YPD and grown over night to saturation. 200 µl of this cell culture were inoculated into 30 ml of SPS and grown until an optical density at 600 nm between 1.2 and 1.6 was reached. The cell suspension was centrifuged for 3 min at 3000 rpm and washed twice with 50 ml of pre-warmed water. Finally, the cell pellet was resuspended in 50 ml SPII. 100 µl of the cell suspension was filled into each of the wells of a microtiter plate. Chemical compound or DMSO was then added to the wells at a 1∶100 dilution; the stock concentration of each compound tested is listed in Table S1. Then the assay plate was covered with a gas permeable sealing membrane and incubated for 5 hours at 30°C with vigorous shaking to keep cells in suspension. 3 µl of cell suspension were then transferred to rectangular -leu and a separate minus -his plates using a multi-channel pipette. Plates were incubated at 30°C. After 24 hours a picture of the -leu plate was taken. After 48 hours a picture of the -his plate was taken and colony density was evaluated with the ImageJ software (National Institutes of Health, USA).

### Growth and Sporulation Inhibitor Screens

To measure growth rates of BY4741 and AD1-9, cells were seeded into microtiter plates at an OD_600_ of 0.065. Chemicals were then added at a 1∶100 dilution, and optical density was measured every 15 min over the course of 24 hours using a Tecan GENios microplate reader (Tecan Systems Inc. San Jose, USA). Average doubling time (AvgG) was calculated by measuring the time the culture took to reach a 5 generation time-point from the starting OD_600_, then divided by the number of generations [38]. The efficiency rate of the his4x/his4B-assay was determined as follows: Colony density on -his plates was measured as Integrated Area (IA) using the ImageJ software. Signals of IA were then averaged for two replicates from two independent experiments. The efficiency rate of the CDA2-based fluorescence assay was determined as follows: The ratio of signal intensities between treatment samples and an average of eight DMSO included on the same assay plate was calculated for every time-point in the fluorescence-based assay. The sum of these ratios was then calculated and divided by the number of measurements.

To calculate the sensitivity scores for the growth assay and the two sporulation assays we first determined the mean and standard deviation (sd) of AvgG or the efficiency rates (as described above) for the eight DMSO controls included in every experiment. For every chemical compound the sensitivity score was then calculated using the mean and sd of the DMSO controls using the formula: (Mean_Value_Compound_ - Mean_Value_DMSO_)/SD_DMSO_. In rare cases the presence of the drug in the media led to an up- or down-ward shift of the baseline fluorescence signal in the sporulation assay (likely due to auto-fluorescence of the compound). To exclude such cases all compounds for which the standard deviation of the ratio of signal intensities between treatment samples and averaged DMSO controls was <0.1 were removed from further analysis. A sensitivity score cutoff of >2 was chosen to identify all chemicals that inhibited vegetative growth. A sensitivity score of >5 (indicating an effect that is very distinct from the one observed in the control samples) was chosen as a cutoff to identify inhibitors in the two sporulation assays, respectively. All compounds and their sensitivity scores are listed in Table S1.

### Microscopy

For Differential interference contrast (DIC) and fluorescence microscopy a microscopy system with the following components was used: Axiovert 40 CFL, AxioCam MRm, LD A-Plan 40x, 1006-595 (Ph2), Axiovision Rel. 4.5 (all parts and software from Zeiss), X-Cite Series 120 (lamp from EXFO). 1 ml cell suspension from sporulating cultures was fixed with 3.7% formaldehyde and incubated for 2 hours at room temperature. The cells were then washed twice in 1 ml of water and resuspended in 1 ml of 70% ethanol. After 30 min of incubation at room temperature cells were washed once with 1 ml water and then resuspended in 200 µl of water mixed with DAPI at a final concentration of 2.5 µg/ml. 5 µl of the cell suspension were then mounted onto a glass slide and analyzed by microscopy.

#### Flow cytometric analysis

1 ml of a growing or sporulating yeast culture (OD_600_ between 1.2 and 1.6; cell density is about 1.5×10^7^) was fixed in 1 ml of Ethanol (70%) and incubated for 60 min at room temperature. The cell suspension was washed twice with 50 mM Na-citrate. 0.3 mg/ml of Ribonuclease A from bovine pancreas were added to the samples followed by incubation at 50°C for 1 h. The cell suspension was washed twice with 50 mM Na-citrate and then mixed with 16 µl of propidium iodide solution. Samples were sonicated for 10 sec at output level 30% using a Branson Digital Sonifier. The following settings were used for FACS analysis using a BD FACSCalibur System: FSC, E00, Log; SSC, 380, Log; FL1-H, 500, Log; FL3-H, 500, Log.

### Total RNA Isolation, cRNA Target Synthesis and GeneChip Hybridization

Cultures of SK1 were grown or sporulated as described in Figure S4A. Samples of 10 ml were then harvested and total RNA was extracted using the RiboPure-Yeast kit (Ambion, catalog no. AM1926). cDNA was synthesized in 10 µl reactions containing 1 µg/µl total RNA, 12.5 ng/µl Oligo(dT)12-18 primer (Invitrogen, catalog no. 18418-012), 15 units/µl SuperScript II (Invitrogen, catalog no. 18064–014), 1× First Strand Buffer, 10 mM DTT, and 10 mM dNTPs (Invitrogen, catalog no. 18427013). After the RNA and primers were denatured for 10 min at 70°C, the remaining reagents were added, and the reaction was incubated at 42°C for 60 min. To remove the RNA template 2 units of RNase H were then added and the mix was incubated at 37°C for 20 min and then at 95°C for 5 min. Quality of total RNA and cRNA was monitored using RNA Nano 6000 chips processed using the 2100 BioAnalyzer (Agilent). 220 µl hybridization cocktail containing heat-fragmented and biotin-labeled cRNA at a concentration of 0.05 µg/µl were injected into GeneChips and incubated at 45°C on a rotator in a Hybridization Oven 640 (Affymetrix) overnight at 60 rpm. The arrays were washed and stained with a streptavidin-phycoerythrin conjugate (SAPE; Molecular Probes). The Gene Chips were processed in a GeneArray Scanner (Agilent) using the default settings. CEL files containing the raw data were computed from DAT array image files using the statistical algorithm implemented in MAS 5.0 (Affymetrix). Log2-transformed raw data were preprocessed (background adjustment, normalization, and summarization of probe sets) by using the Robust Multiarray Analysis (RMA) package from BioConductor. CEL feature-level data files are available via the EBI ArrayExpress public data repository at under accession number E-MEXP-2522.

### Homozygous Deletion Profiling

For genome-wide fitness profiles the complete set of homozygous deletion strains in the diploid BY4743 background was inoculated in 70 ml YPD and grown for 12 hours. The saturated cell suspension was then transferred into SPII, after washing twice with distilled water. This culture was split into two halves, and 100 µM tripelennamine was added to one half; the other half served as a “no drug” control. After 60 hours of incubation in SPII 200 µl of the cell suspension were transferred into 20 ml of YPD and grown for 16 hours. Samples were processed as described previously [39]. Log2-transformed and quantile normalized microarray data were analyzed using the Significance Analysis of Microarrays (SAM) software [57] and genes that showed significant changes in TA compared to the “no drug” control were identified using a cutoff of delta  = 1. CEL feature-level data files are available at ArrayExpress under accession number E-MEXP-2535.

### Metabolome Analyses

Sampling for intracellular metabolites was carried out by filtering 5 mL of culture medium on a polyamide membrane (pore size 0.45 µm, Sartorius), rinsing the cells on filter using 10 mL water containing only the corresponding carbon source, and quenching the cells’ metabolism in 80°C hot ethanol (75%). Processing of the samples was done as described earlier [58], [59]. Metabolites were quantified using LC-MS: Liquid anion exchange chromatography was performed on an ICS-3000 system from Dionex (Sunnyvale, USA) equipped with an automatic eluent (KOH) generator system (RFIC, Dionex), and an autosampler (AS50, Dionex) holding the samples at 4°C. Analytes were separated on an IonPac AS11 (250×2 mm, Dionex) column protected by an AG11 (50×2 mm, Dionex) pre-column. Column temperature was held at 25°C, flow rate was fixed at 0.25 ml/min, and analytes were eluted applying the KOH gradient described earlier [60]. Injected sample volume was 15 µl. For background reduction, an ASRS ultra II (2 mm) anion suppressor was used. Analytes were quantified using a photo diode array detector (Ultimate 3000, Dionex), a conductivity detector (part of ICS-3000, Dionex) and a mass-sensitive detector (MSQ Plus, Thermo) running in ESI mode (nitrogen pressure was 90 psi, capillary voltage was 3.5 kV, probe temperature was 450°C).

## Supporting Information

Figure S1 **Small molecules that inhibit vegetative growth.** (A) Examples of growth curves of the wild-type BY4741 strain (control, left plot) and the drug-efflux pump deficient strain (AD1-9, right plot) grown in rich media in the presence of different organic compounds from the NIH clinical collection. Optical density of both strains was measured every 15 min over a period of 20 hours grown in the presence of DMSO, Raclopride, Fluvoxamine, Duloxetine, or Clotrimazole (depicted as black, red, green, dark blue, and light blue curves, respectively). The concentration of DMSO was 1%; all other compounds were tested at 100 µM. (B) Scatterplot of growth rates (measured as AvgG, see Methods) of AD1-9 and BY4741 (control) grown in rich media and each of 446 compounds in the NIH clinical collection. Higher values of AvgG indicate reduced growth rates. In cases where vegetative growth was completely suppressed we assigned a value of 20. As expected, in many cases the pump-deficient AD1-9 strain was more inhibited by a compound than the BY4741 control strain.
(TIF)

Figure S2 **Tripelennamine does not inhibit growth of wild-type yeast.** Growth curve analysis of the wild-type BY4741 strain grown in rich media (left plot) or minimal media (right plot) in the presence or absence of tripelennamine (see legend). Optical density was measured every 15 min over a period of 20 hours. 0, 100, or 200 µM of tripelennamine was added to the cultures at the beginning of the experiment, depicted as black, red, and green curves, respectively. As expected, cultures grew at a lower rate in minimal media when compared to rich media. No difference in growth rate was, however, observed in the absence or presence of tripelennamine.
(TIF)

Figure S3 **Timing of triplennamine-mediated inhibition of sporulation.** Two types of time-course experiments were performed: In the first, 100 µM tripelennamine (TA) was added at 0, 3, 6, 9, and 12 hours after induction of sporulation (‘addition’, black curve). In the second 100 µM TA was added to the culture at the onset of sporulation and then washed out of the media after 0, 3, 6, 9, and 12 hours (‘wash-out’, red curve). The fraction of spores in each culture was determined after 24 hours by microscopy. A total of 100 cells were counted for every condition.
(TIF)

Figure S4 **Tripelennamine induces genes involved in nutrient catabolism and multidrug resistance.** (A) Flow chart of the experimental protocol. Samples that were analyzed with microarrays are marked in bold. N: “no drug” control; T: tripelennamine (100 µM). (B) Heatmap and dendrogram depicting hierarchical clustering of expression profiling data. Loci with an at least 2-fold higher expression in tripelennamine-treated samples (T4 and T8) compared to the “no drug” control (N4 and N8) at the 4 and the 8 hour time-point are shown. Sample names are as indicated in (A). A color-coded scale for log2-transformed expression values is given at the bottom. Genes involved in glycolysis/gluconeogenesis or amino acid uptake/metabolism are marked with black dots in the GL and AA columns, respectively. (C) Expression patterns of the drug-efflux pump genes *PDR5* and *SNQ2*. Log2-transformed fluorescence signals are plotted on the y-axis and are graphed versus samples harvested in rich media and pre-sporulation media (in the absence of tripelennamine), or total time (4 and 8 hours) the cultures spent in sporulation media in the absence (black curve) or presence (red curve) of tripelennamine.
(TIF)

Figure S5 **Yeast strains heterozygous in the NEO1 locus are hypersensitized by tripelennamine.** Growth curves of a wild-type (left panel) and a *neo1*Δ*/NEO1* heterozygous deletion strain in the SK1 strain background (right panel) grown in the presence of various concentrations of tripelennamine (indicated in the legend). Optical density of both strains was measured every 15 min over a period of 20 hours.
(TIF)

Table S1 **Complete set of screening data for 446 organic compounds.** The names, stock concentrations, chemical database identifier and sensitivity scores calculated for the growth assay, CDA2 assay, and the his4 assay are listed for the 446 compounds in the NIH clinical collection (NCC). Chemicals are shown in order of their position in the NCC library.
(XLS)

Table S2 **Complete set of metabolome data for tripelennamine-treated sporulating cells.** Concentrations (given in [µmol/g of dry weight]) of 37 metabolites involved in glycolysis, TCA and glyoxylate cycle, nucleotide metabolism, and reserve carbohydrate metabolism were quantified by IC-MS analyses (see Methods). Data for duplicate samples (sample 1 and sample 2, respectively), for cells sporulated for 0, 5, 9, and 24 hours in the presence or absence of tripelennamine (+TA and –TA, respectively) are listed. Missing values are indicated by ‘NA’.
(XLS)

## References

[1] Mendola P, Messer LC, Rappazzo K (2008) Science linking environmental contaminant exposures with fertility and reproductive health impacts in the adult female. Fertil Steril 89: e81–94. 18308071 10.1016/j.fertnstert.2007.12.036

[2] Thonneau P, Bujan L, Multigner L, Mieusset R (1998) Occupational heat exposure and male fertility: a review. Hum Reprod 13: 2122–2125. 9756281 10.1093/humrep/13.8.2122

[3] Schlecht U, Demougin P, Koch R, Hermida L, Wiederkehr C, et al (2004) Expression profiling of mammalian male meiosis and gametogenesis identifies novel candidate genes for roles in the regulation of fertility. Mol Biol Cell 15: 1031–1043. 14718556 10.1091/mbc.E03-10-0762 PMC363067

[4] Schlecht U, Primig M (2003) Mining meiosis and gametogenesis with DNA microarrays. Reproduction 125: 447–456. 12683916 10.1530/rep.0.1250447

[5] Chalmel F, Rolland AD, Niederhauser-Wiederkehr C, Chung SS, Demougin P, et al (2007) The conserved transcriptome in human and rodent male gametogenesis. Proc Natl Acad Sci U S A 104: 8346–8351. 17483452 10.1073/pnas.0701883104 PMC1864911

[6] Honigberg SM, Purnapatre K (2003) Signal pathway integration in the switch from the mitotic cell cycle to meiosis in yeast. J Cell Sci 116: 2137–2147. 12730290 10.1242/jcs.00460

[7] Primig M, Williams RM, Winzeler EA, Tevzadze GG, Conway AR, et al (2000) The core meiotic transcriptome in budding yeasts. Nat Genet 26: 415–423. 11101837 10.1038/82539

[8] Chu S, DeRisi J, Eisen M, Mulholland J, Botstein D, et al (1998) The transcriptional program of sporulation in budding yeast. Science 282: 699–705. 9784122 10.1126/science.282.5389.699

[9] Engebrecht JA, Voelkel-Meiman K, Roeder GS (1991) Meiosis-specific RNA splicing in yeast. Cell 66: 1257–1268. 1840507 10.1016/0092-8674(91)90047-3

[10] Juneau K, Palm C, Miranda M, Davis RW (2007) High-density yeast-tiling array reveals previously undiscovered introns and extensive regulation of meiotic splicing. Proc Natl Acad Sci U S A 104: 1522–1527. 17244705 10.1073/pnas.0610354104 PMC1780280

[11] Surosky RT, Esposito RE (1992) Early meiotic transcripts are highly unstable in Saccharomyces cerevisiae. Mol Cell Biol 12: 3948–3958. 1508196 10.1128/mcb.12.9.3948-3958.1992 PMC360277

[12] Guttmann-Raviv N, Martin S, Kassir Y (2002) Ime2, a meiosis-specific kinase in yeast, is required for destabilization of its transcriptional activator, Ime1. Mol Cell Biol 22: 2047–2056. 11884593 10.1128/MCB.22.7.2047-2056.2002 PMC133691

[13] Mallory MJ, Cooper KF, Strich R (2007) Meiosis-specific destruction of the Ume6p repressor by the Cdc20-directed APC/C. Mol Cell 27: 951–961. 17889668 10.1016/j.molcel.2007.08.019 PMC2034308

[14] Esposito RE (2006) Meiosis and spore development in Landmark papers in Yeast Biology. Cold Spring Harbor Laboratory Press. 157–192.

[15] Kassir Y, Adir N, Boger-Nadjar E, Raviv NG, Rubin-Bejerano I, et al (2003) Transcriptional regulation of meiosis in budding yeast. Int Rev Cytol 224: 111–171. 12722950 10.1016/s0074-7696(05)24004-4

[16] Kane SM, Roth R (1974) Carbohydrate metabolism during ascospore development in yeast. J Bacteriol 118: 8–14. 4595206 10.1128/jb.118.1.8-14.1974 PMC246633

[17] Neiman AM (2005) Ascospore formation in the yeast Saccharomyces cerevisiae. Microbiol Mol Biol Rev 69: 565–584. 16339736 10.1128/MMBR.69.4.565-584.2005 PMC1306807

[18] Betz H, Weiser U (1976) Protein degradation during yeast sporulation. Enzyme and cytochrome patterns. Eur J Biochem 70: 385–395. 188644 10.1111/j.1432-1033.1976.tb11028.x

[19] Miller JJ (1963) The metabolism of yeast sporulation: V. Stimulation and inhibition of sporulation and growth by nitrogen compounds. Can J Microbiol 9: 259–277.

[20] Sora S, Crippa M, Lucchini G (1983) Disomic and diploid meiotic products in Saccharomyces cerevisiae. Effect of vincristine, vinblastine, adriamycin, bleomycin, mitomycin C and cyclophosphamide. Mutat Res 107: 249–264. 6191212 10.1016/0027-5107(83)90167-7

[21] Christodoulidou A, Bouriotis V, Thireos G (1996) Two sporulation-specific chitin deacetylase-encoding genes are required for the ascospore wall rigidity of Saccharomyces cerevisiae. J Biol Chem 271: 31420–31425. 8940152 10.1074/jbc.271.49.31420

[22] Schlecht U, Erb I, Demougin P, Robine N, Borde V, et al (2008) Genome-wide expression profiling, in vivo DNA binding analysis, and probabilistic motif prediction reveal novel Abf1 target genes during fermentation, respiration, and sporulation in yeast. Mol Biol Cell 19: 2193–2207. 18305101 10.1091/mbc.E07-12-1242 PMC2366881

[23] Kassir Y, Granot D, Simchen G (1988) IME1, a positive regulator gene of meiosis in S. cerevisiae. Cell 52: 853–862. 3280136 10.1016/0092-8674(88)90427-8

[24] Cao L, Alani E, Kleckner N (1990) A pathway for generation and processing of double-strand breaks during meiotic recombination in S. cerevisiae. Cell 61: 1089–1101. 2190690 10.1016/0092-8674(90)90072-m

[25] Storlazzi A, Xu L, Cao L, Kleckner N (1995) Crossover and noncrossover recombination during meiosis: timing and pathway relationships. Proc Natl Acad Sci U S A 92: 8512–8516. 7667321 10.1073/pnas.92.18.8512 PMC41187

[26] Fan Q, Xu F, Petes TD (1995) Meiosis-specific double-strand DNA breaks at the HIS4 recombination hot spot in the yeast Saccharomyces cerevisiae: control in cis and trans. Mol Cell Biol 15: 1679–1688. 7862159 10.1128/mcb.15.3.1679 PMC230392

[27] Esposito RE, Esposito MS (1974) Genetic recombination and commitment to meiosis in Saccharomyces. Proc Natl Acad Sci U S A 71: 3172–3176. 4606582 10.1073/pnas.71.8.3172 PMC388644

[28] Brachmann CB, Davies A, Cost GJ, Caputo E, Li J, et al (1998) Designer deletion strains derived from Saccharomyces cerevisiae S288C: a useful set of strains and plasmids for PCR-mediated gene disruption and other applications. Yeast 14: 115–132. 9483801 10.1002/(SICI)1097-0061(19980130)14:2<115::AID-YEA204>3.0.CO;2-2

[29] Rogers B, Decottignies A, Kolaczkowski M, Carvajal E, Balzi E, et al (2001) The pleitropic drug ABC transporters from Saccharomyces cerevisiae. J Mol Microbiol Biotechnol 3: 207–214. 11321575

[30] Vershon AK, Pierce M (2000) Transcriptional regulation of meiosis in yeast. Curr Opin Cell Biol 12: 334–339. 10801467 10.1016/s0955-0674(00)00104-6

[31] Briza P, Bogengruber E, Thur A, Rutzler M, Munsterkotter M, et al (2002) Systematic analysis of sporulation phenotypes in 624 non-lethal homozygous deletion strains of Saccharomyces cerevisiae. Yeast 19: 403–422. 11921089 10.1002/yea.843

[32] Deutschbauer AM, Williams RM, Chu AM, Davis RW (2002) Parallel phenotypic analysis of sporulation and postgermination growth in Saccharomyces cerevisiae. Proc Natl Acad Sci U S A 99: 15530–15535. 12432101 10.1073/pnas.202604399 PMC137751

[33] Enyenihi AH, Saunders WS (2003) Large-scale functional genomic analysis of sporulation and meiosis in Saccharomyces cerevisiae. Genetics 163: 47–54. 12586695 10.1093/genetics/163.1.47 PMC1462418

[34] Rabitsch KP, Toth A, Galova M, Schleiffer A, Schaffner G, et al (2001) A screen for genes required for meiosis and spore formation based on whole-genome expression. Curr Biol 11: 1001–1009. 11470404 10.1016/s0960-9822(01)00274-3

[35] Esposito MS, Esposito RE, Arnaud M, Halvorson HO (1969) Acetate utilization and macromolecular synthesis during sporulation of yeast. J Bacteriol 100: 180–186. 5344095 10.1128/jb.100.1.180-186.1969 PMC315375

[36] Tenreiro S, Rosa PC, Viegas CA, Sa-Correia I (2000) Expression of the AZR1 gene (ORF YGR224w), encoding a plasma membrane transporter of the major facilitator superfamily, is required for adaptation to acetic acid and resistance to azoles in Saccharomyces cerevisiae. Yeast 16: 1469–1481. 11113970 10.1002/1097-0061(200012)16:16<1469::AID-YEA640>3.0.CO;2-A

[37] Hillenmeyer ME, Fung E, Wildenhain J, Pierce SE, Hoon S, et al (2008) The chemical genomic portrait of yeast: uncovering a phenotype for all genes. Science 320: 362–365. 18420932 10.1126/science.1150021 PMC2794835

[38] Lee W, St Onge RP, Proctor M, Flaherty P, Jordan MI, et al (2005) Genome-wide requirements for resistance to functionally distinct DNA-damaging agents. PLoS Genet 1: e24. 16121259 10.1371/journal.pgen.0010024 PMC1189734

[39] Giaever G, Chu AM, Ni L, Connelly C, Riles L, et al (2002) Functional profiling of the Saccharomyces cerevisiae genome. Nature 418: 387–391. 12140549 10.1038/nature00935

[40] Rainey MM, Korostyshevsky D, Lee S, Perlstein EO (2010) The antidepressant sertraline targets intracellular vesiculogenic membranes in yeast. Genetics 185: 1221–1233. 20457874 10.1534/genetics.110.117846 PMC2927751

[41] Paulusma CC, Oude Elferink RP (2005) The type 4 subfamily of P-type ATPases, putative aminophospholipid translocases with a role in human disease. Biochim Biophys Acta 1741: 11–24. 15919184 10.1016/j.bbadis.2005.04.006

[42] Brett CL, Kallay L, Hua Z, Green R, Chyou A, et al (2011) Genome-wide analysis reveals the vacuolar pH-stat of Saccharomyces cerevisiae. PLoS One 6: e17619. 21423800 10.1371/journal.pone.0017619 PMC3056714

[43] Anderson N, Borlak J (2006) Drug-induced phospholipidosis. FEBS Lett 580: 5533–5540. 16979167 10.1016/j.febslet.2006.08.061

[44] Kim J, Klionsky DJ (2000) Autophagy, cytoplasm-to-vacuole targeting pathway, and pexophagy in yeast and mammalian cells. Annu Rev Biochem 69: 303–342. 10966461 10.1146/annurev.biochem.69.1.303

[45] Klionsky DJ (2007) Autophagy: from phenomenology to molecular understanding in less than a decade. Nat Rev Mol Cell Biol 8: 931–937. 17712358 10.1038/nrm2245

[46] Poole B, Ohkuma S (1981) Effect of weak bases on the intralysosomal pH in mouse peritoneal macrophages. J Cell Biol 90: 665–669. 6169733 10.1083/jcb.90.3.665 PMC2111912

[47] Glaumann H, Ahlberg J (1987) Comparison of different autophagic vacuoles with regard to ultrastructure, enzymatic composition, and degradation capacity–formation of crinosomes. Exp Mol Pathol 47: 346–362. 3678466 10.1016/0014-4800(87)90018-9

[48] Takeshige K, Baba M, Tsuboi S, Noda T, Ohsumi Y (1992) Autophagy in yeast demonstrated with proteinase-deficient mutants and conditions for its induction. J Cell Biol 119: 301–311. 1400575 10.1083/jcb.119.2.301 PMC2289660

[49] Onodera J, Ohsumi Y (2005) Autophagy is required for maintenance of amino acid levels and protein synthesis under nitrogen starvation. J Biol Chem 280: 31582–31586. 16027116 10.1074/jbc.M506736200

[50] Noda T, Kim J, Huang WP, Baba M, Tokunaga C, et al (2000) Apg9p/Cvt7p is an integral membrane protein required for transport vesicle formation in the Cvt and autophagy pathways. J Cell Biol 148: 465–480. 10662773 10.1083/jcb.148.3.465 PMC2174799

[51] Shintani T, Suzuki K, Kamada Y, Noda T, Ohsumi Y (2001) Apg2p functions in autophagosome formation on the perivacuolar structure. J Biol Chem 276: 30452–30460. 11382761 10.1074/jbc.M102346200

[52] Barth H, Meiling-Wesse K, Epple UD, Thumm M (2001) Autophagy and the cytoplasm to vacuole targeting pathway both require Aut10p. FEBS Lett 508: 23–28. 11707261 10.1016/s0014-5793(01)03016-2

[53] Piekarska I, Kucharczyk R, Mickowska B, Rytka J, Rempola B (2010) Mutants of the Saccharomyces cerevisiae VPS genes CCZ1 and YPT7 are blocked in different stages of sporulation. Eur J Cell Biol 89: 780–787. 20709422 10.1016/j.ejcb.2010.06.009

[54] Wicky S, Schwarz H, Singer-Kruger B (2004) Molecular interactions of yeast Neo1p, an essential member of the Drs2 family of aminophospholipid translocases, and its role in membrane trafficking within the endomembrane system. Mol Cell Biol 24: 7402–7418. 15314152 10.1128/MCB.24.17.7402-7418.2004 PMC507011

[55] Rose MD, Winston F, Hieter P (1990) Methods in Yeast Genetics: A Laboratory Course Manual. Cold Spring Harbor Laboratory Press.

[56] Ito H, Fukuda Y, Murata K, Kimura A (1983) Transformation of intact yeast cells treated with alkali cations. J Bacteriol 153: 163–168. 6336730 10.1128/jb.153.1.163-168.1983 PMC217353

[57] Tusher VG, Tibshirani R, Chu G (2001) Significance analysis of microarrays applied to the ionizing radiation response. Proc Natl Acad Sci U S A 98: 5116–5121. 11309499 10.1073/pnas.091062498 PMC33173

[58] Gonzalez B, Francois J, Renaud M (1997) A rapid and reliable method for metabolite extraction in yeast using boiling buffered ethanol. Yeast 13: 1347–1355. 9392079 10.1002/(SICI)1097-0061(199711)13:14<1347::AID-YEA176>3.0.CO;2-O

[59] Loret MO, Pedersen L, Francois J (2007) Revised procedures for yeast metabolites extraction: application to a glucose pulse to carbon-limited yeast cultures, which reveals a transient activation of the purine salvage pathway. Yeast 24: 47–60. 17192850 10.1002/yea.1435

[60] Groussac E, Ortiz M, Francois J (2000) Improved protocols for quantitative determination of metabolites from biological samples using high performance ionic-exchange chromatography with conductimetric and pulsed amperometric detection. Enzyme Microb Technol 26: 715–723. 10862877 10.1016/s0141-0229(00)00163-0

