# Supplementary figures and images for: Cationic Amphiphilic Drugs Are Potent Inhibitors of Yeast Sporulation
Source: PLoS One. 2012 Aug 8;7(8):e42853. doi: 10.1371/journal.pone.0042853 (PMC3414501; doi:10.1371/journal.pone.0042853)

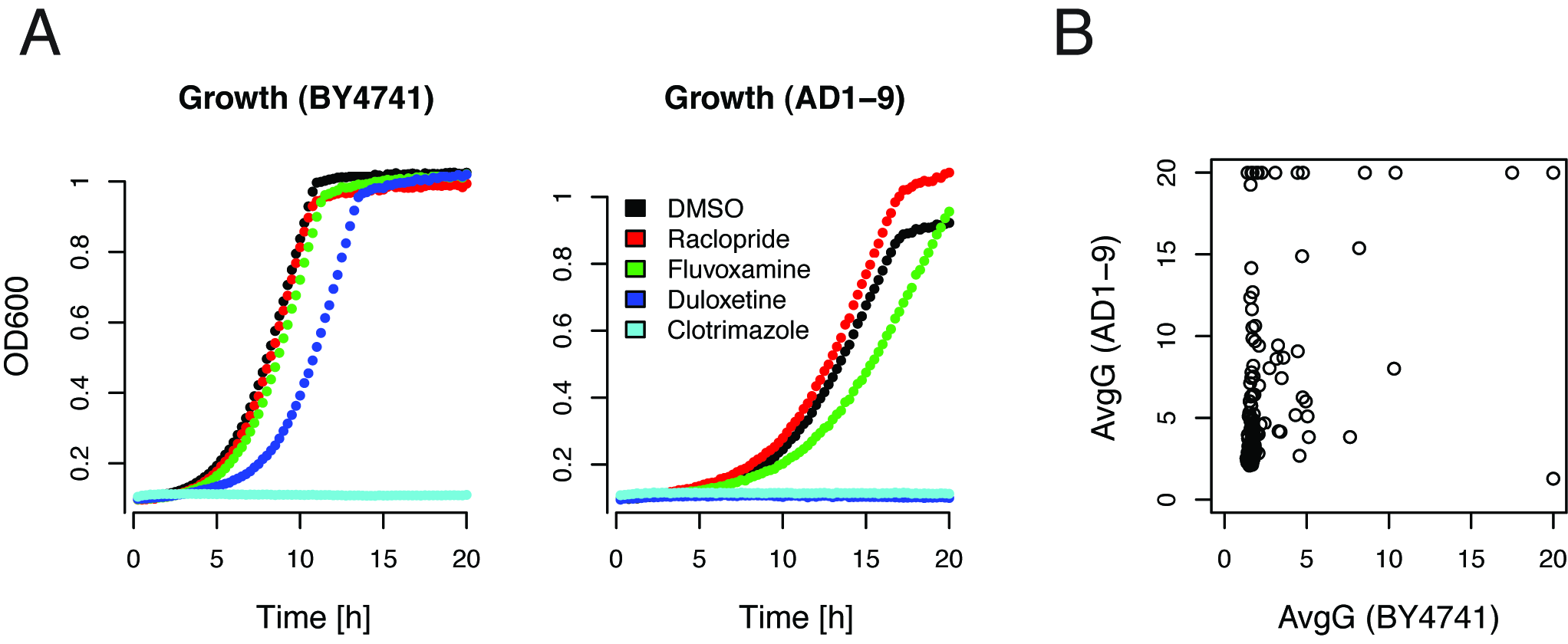

Supplement: Figure S1 — Small molecules that inhibit vegetative growth. (A) Examples of growth curves of the wild-type BY4741 strain (control, left plot) and the drug-efflux pump deficient strain (AD1-9, right plot) grown in rich media in the presence of different organic compounds from the NIH clinical collection. Optical density of both strains was measured every 15 min over a period of 20 hours grown in the presence of DMSO, Raclopride, Fluvoxamine, Duloxetine, or Clotrimazole (depicted as black, red, green, dark blue, and light blue curves, respectively). The concentration of DMSO was 1%; all other compounds were tested at 100 µM. (B) Scatterplot of growth rates (measured as AvgG, see Methods) of AD1-9 and BY4741 (control) grown in rich media and each of 446 compounds in the NIH clinical collection. Higher values of AvgG indicate reduced growth rates. In cases where vegetative growth was completely suppressed we assigned a value of 20. As expected, in many cases the pump-deficient AD1-9 strain was more inhibited by a compound than the BY4741 control strain. (TIF) [file pone.0042853.s001.tif]

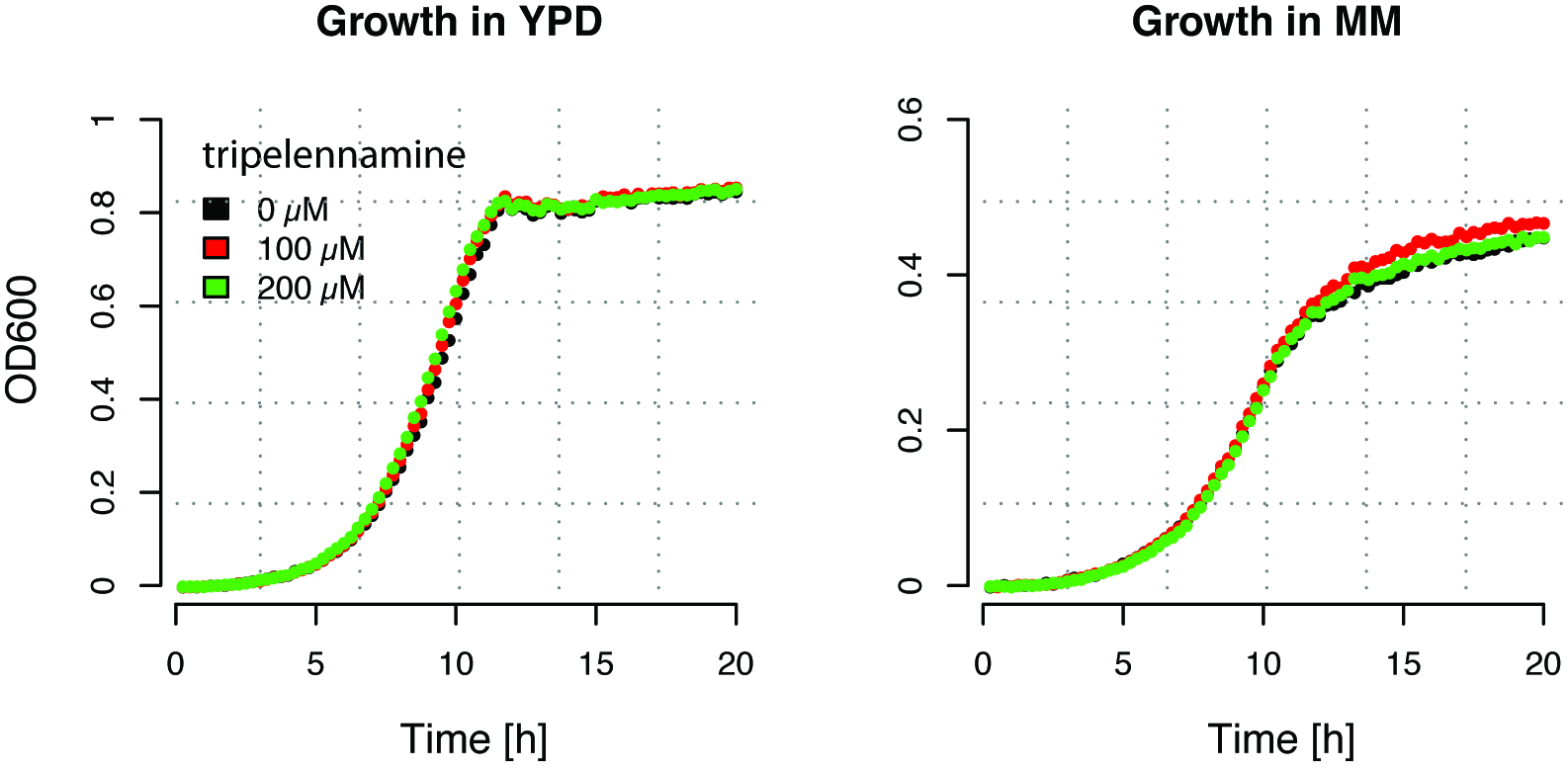

Supplement: Figure S2 — Tripelennamine does not inhibit growth of wild-type yeast. Growth curve analysis of the wild-type BY4741 strain grown in rich media (left plot) or minimal media (right plot) in the presence or absence of tripelennamine (see legend). Optical density was measured every 15 min over a period of 20 hours. 0, 100, or 200 µM of tripelennamine was added to the cultures at the beginning of the experiment, depicted as black, red, and green curves, respectively. As expected, cultures grew at a lower rate in minimal media when compared to rich media. No difference in growth rate was, however, observed in the absence or presence of tripelennamine. (TIF) [file pone.0042853.s002.tif]

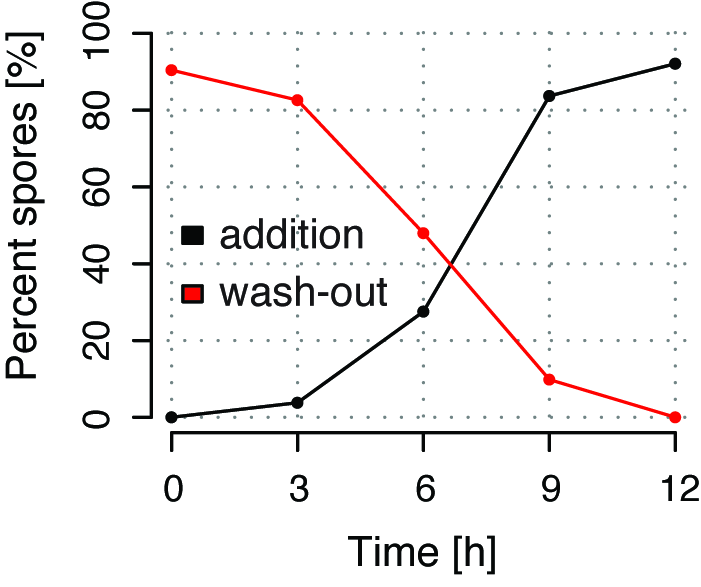

Supplement: Figure S3 — Timing of triplennamine-mediated inhibition of sporulation. Two types of time-course experiments were performed: In the first, 100 µM tripelennamine (TA) was added at 0, 3, 6, 9, and 12 hours after induction of sporulation (‘addition’, black curve). In the second 100 µM TA was added to the culture at the onset of sporulation and then washed out of the media after 0, 3, 6, 9, and 12 hours (‘wash-out’, red curve). The fraction of spores in each culture was determined after 24 hours by microscopy. A total of 100 cells were counted for every condition. (TIF) [file pone.0042853.s003.tif]

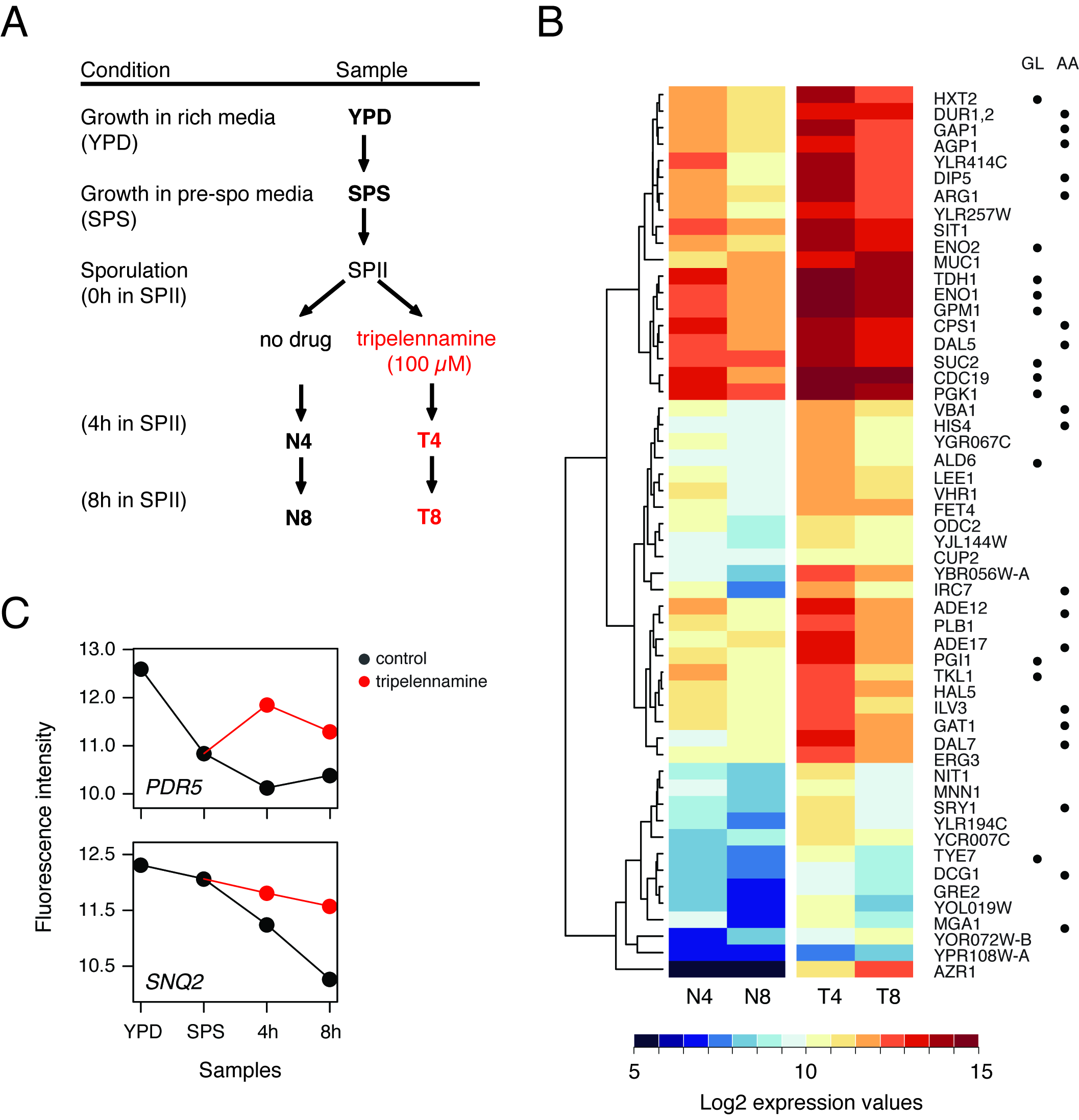

Supplement: Figure S4 — Tripelennamine induces genes involved in nutrient catabolism and multidrug resistance. (A) Flow chart of the experimental protocol. Samples that were analyzed with microarrays are marked in bold. N: “no drug” control; T: tripelennamine (100 µM). (B) Heatmap and dendrogram depicting hierarchical clustering of expression profiling data. Loci with an at least 2-fold higher expression in tripelennamine-treated samples (T4 and T8) compared to the “no drug” control (N4 and N8) at the 4 and the 8 hour time-point are shown. Sample names are as indicated in (A). A color-coded scale for log2-transformed expression values is given at the bottom. Genes involved in glycolysis/gluconeogenesis or amino acid uptake/metabolism are marked with black dots in the GL and AA columns, respectively. (C) Expression patterns of the drug-efflux pump genes PDR5 and SNQ2. Log2-transformed fluorescence signals are plotted on the y-axis and are graphed versus samples harvested in rich media and pre-sporulation media (in the absence of tripelennamine), or total time (4 and 8 hours) the cultures spent in sporulation media in the absence (black curve) or presence (red curve) of tripelennamine. (TIF) [file pone.0042853.s004.tif]

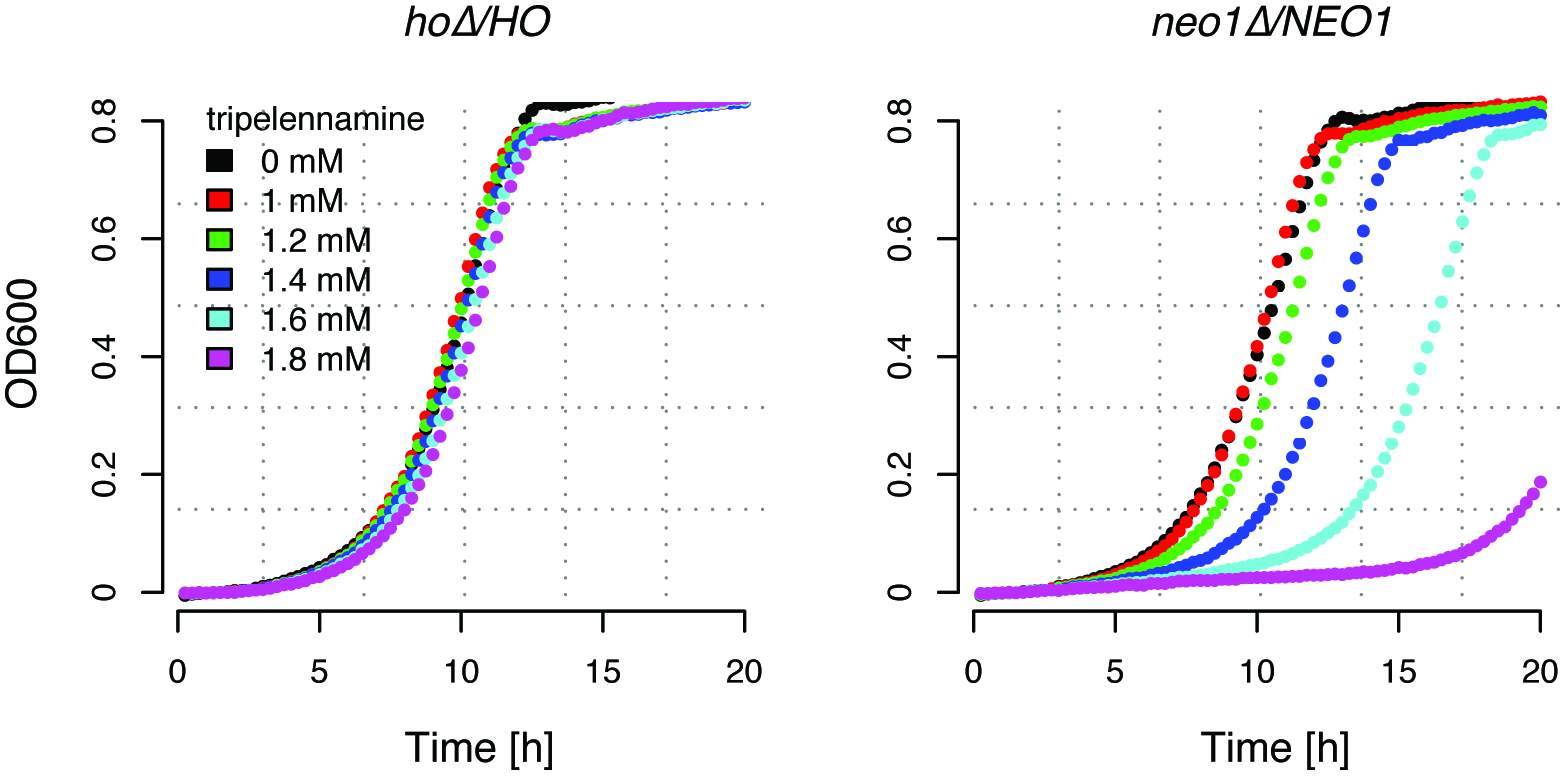

Supplement: Figure S5 — Yeast strains heterozygous in the NEO1 locus are hypersensitized by tripelennamine. Growth curves of a wild-type (left panel) and a neo1Δ/NEO1 heterozygous deletion strain in the SK1 strain background (right panel) grown in the presence of various concentrations of tripelennamine (indicated in the legend). Optical density of both strains was measured every 15 min over a period of 20 hours. (TIF) [file pone.0042853.s005.tif]
